# Dynamic regulation of nuclear architecture and mechanics—a rheostatic role for the nucleus in tailoring cellular mechanosensitivity

**DOI:** 10.1080/19491034.2017.1285988

**Published:** 2017-02-02

**Authors:** Stephen D. Thorpe, David A. Lee

**Affiliations:** Institute of Bioengineering, School of Engineering and Materials Science, Queen Mary University of London, London, UK

**Keywords:** chromatin, differentiation, embryonic stem cell, lamin A/C, LINC complex, mechanobiology, mechanotransduction, mesenchymal stem cell, nucleus

## Abstract

Nuclear architecture, a function of both chromatin and nucleoskeleton structure, is known to change with stem cell differentiation and differs between various somatic cell types. These changes in nuclear architecture are associated with the regulation of gene expression and genome function in a cell-type specific manner. Biophysical stimuli are known effectors of differentiation and also elicit stimuli-specific changes in nuclear architecture. This occurs via the process of mechanotransduction whereby extracellular mechanical forces activate several well characterized signaling cascades of cytoplasmic origin, and potentially some recently elucidated signaling cascades originating in the nucleus. Recent work has demonstrated changes in nuclear mechanics both with pluripotency state in embryonic stem cells, and with differentiation progression in adult mesenchymal stem cells. This review explores the interplay between cytoplasmic and nuclear mechanosensitivity, highlighting a role for the nucleus as a rheostat in tuning the cellular mechano-response.

## Introduction

Mechanical forces influence the growth and form of practically all tissues in the human body. To survive routine physical exertion and its associated stresses, load bearing tissues such as bone and cartilage are stiff, while some non-load bearing tissues such as brain and marrow are effectively shielded from external mechanical loads. Tissue level deformations are transferred via the extracellular matrix (ECM) to cells residing within. The nature of the cell's interaction with the ECM determines the extent of deformation experienced; which may be damped or amplified.^1^ Likewise, it is the nature of nuclear interactions with the cytoskeleton which dictates the extent of nuclear deformation in response to a given cellular strain.^2^

Mechanotransduction is the conversion of mechanical stimuli into an intracellular biochemical response. To date, the majority of mechanotransduction research has focused on the perception of mechanical forces at and across the cell membrane to induce signaling pathways originating in the cytoplasm.^2,3^ However, a spate of recent research has identified various mechanisms through which the nuclear envelope and its associated proteins directly respond to mechanical perturbations; summarised in some recent reviews.^4-6^ The origin of the mechanotransduction response within the cell likely directs the nature of the biochemical response in a pathway specific manner. This review provides an overview of the current understanding of the role of the nucleus and nuclear envelope in mechanosensing and mechanotransduction. We then address the concept that the interplay in connectivity and mechanical properties between the nucleus and cytoplasm provides a mechanism to direct the origin of mechanotransduction within the cell to tailor mechanosensitivity.

## Physically connecting the extracellular matrix with the nucleus

### Force transmission and mechanotransduction at the cell membrane

**Figure 1.**
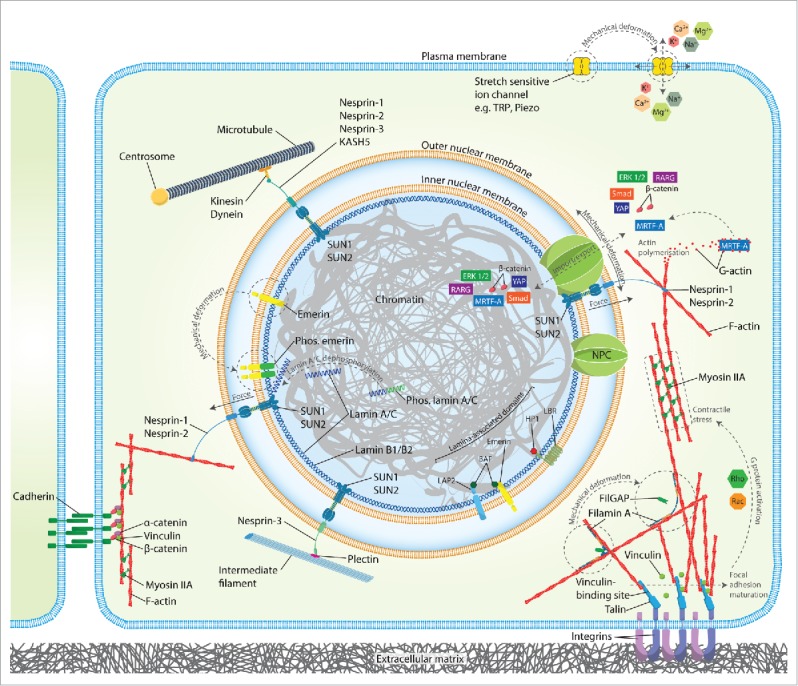
Force transmission and mechanotransduction from the extracellular matrix (ECM) to the nucleus. Schematic illustration of the various connections facilitating force transmission and mechanotransduction across the cell's plasma membrane and nuclear envelope. External forces are transmitted across the plasma membrane via integrins attached to ECM, or cadherins at cell-cell junctions. These forces induce a range of local cytoplasmic mechanotransduction pathways. Additionally, force propagation through the cytoskeleton to the LINC complex facilitates the activation of additional mechanotransduction pathways in the nucleus. Deformation of both plasma and nuclear membranes changes ion channel and nuclear pore complex (NPC) permeability facilitating import and export of various signaling molecules. Mechano-responses are indicated in gray with dashed arrows. It is not possible to include every putative mechanotransduction pathway in this figure and only those covered in this review are included.

The cell's interaction with its environment, be it bound to ECM, a neighboring cell, or suspended in fluid, dictates both the nature of an applied mechanical stimulus, and the cellular machinery involved in subsequent mechanotransduction. At the cell surface, signaling is typically induced by mechanical forces which deform the plasma membrane along with its associated membrane-bound proteins and cytoskeletal elements (Fig. 1).^3,7^ There are various structures at the plasma membrane with mechanotransduction roles specific to aspects of the mechanical environment. Cell-ECM interactions are typically mediated through focal adhesion-based integrin adhesions,^8,9^ while cadherin-based adhesions mediate cell-cell junctions.^10^ Mechanosensitive ion channels,^11,12^ G-protein coupled receptors,^13^ and changes in lipid microdomains^14,15^ also act to convey mechanical signals across the plasma membrane.

Mechano-sensation at the plasma membrane leads to downstream nucleocytoplasmic shuttling of various transcription regulators. Canonical Wnt/β-catenin signaling is one such pathway.^16^ It involves the translocation of stabilised β-catenin to the nucleus where it associates with transcription factors including T cell factor (TCF) and lymphoid enhancer-binding factor (LEF) to regulate the transcription of target genes prominently associated with differentiation and proliferation.^17^ The Hippo pathway tumor suppressor proteins, Yes-associated protein (YAP) and transcriptional coactivator with PDZ-binding motif (TAZ), have recently been found to mediate mechanical cues via their translocation to the nucleus in response to Rho and actomyosin tension.^18,19^ Crosstalk between these factors adds a further layer of complexity and fine-tuning to these signaling pathways. For example, a context-dependent switch in chemo/mechanotransduction has been observed via crosstalk between the Rho pathway effector, myocardin-related transcription factor (MRTF-A; also known as MAL or MKL1), TAZ, and transforming growth factor-β (TGF-β)-regulated Smad nuclear translocation.^20^

### Force transmission through the nuclear membrane

Mechanically induced gene expression changes are often brought about through indirect biochemical signaling initiated at the cell surface or through cytoskeletal stiffening. However, force can also be transmitted from the plasma membrane, via the cytoskeleton, directly to the nucleus, allowing the nuclear membrane and its associated proteins to respond directly (Fig. 1). This process does not require biochemical signaling and can occur over much shorter time scales (∼1 ms versus 5–10 s).^2^ To sense extracellular force, the nucleus must be physically coupled to membrane bound adhesive complexes. Maniotis et al. first demonstrated the existence of a mechanical link between the plasma membrane and the nucleus.^21^ Integrin-bound microbeads or micropipettes were perturbed resulting in cytoskeletal filament reorientation, nuclei distortion and nucleoli redistribution. These findings suggest that an externally applied force could not only deform the nucleus, but induce reorganisation of its genomic contents, potentially regulating gene expression. This force propagation is mediated by both intermediate filaments and F-actin, and requires some cytoskeletal prestress.^21,22^

A specialized anchoring structure exists at the nuclear envelope known as the linker of nucleoskeleton and cytoskeleton (LINC) complex which contains nesprins, sun and lamin proteins,^23^ and provides a functional link between the support structures of the cytoplasmic and nucleoplasmic compartments (Fig. 1).^24^ Nesprin-1 and nesprin-2 on the outer nuclear membrane (ONM) connect to actin filaments,^25,26^ in addition to dynein and kinesin, motor proteins of the microtubule network.^27,28^ Nesprin-3 binds plectin which connects to networks of intermediate filaments.^29^ On the inner nuclear membrane (INM), Sad1 and UNC84 (SUN) domain proteins, Sun1, Sun2 and Sun3 interact with the nuclear pore complex,^30^ lamin A,^31,32^ and chromatin^33^ in the nucleoplasm. Nesprins span the ONM, and via a luminal Klarsicht/Anc-1/Syne-1 homology (KASH) domain, interact with SUN domains; establishing the LINC complex and maintaining the perinuclear space.^34^

On the nucleoplasmic side of the INM lies the lamina. This structural network of intermediate filaments is composed largely of A and B type lamins and the proteins that associate with them, the lamin-associated proteins and lamin receptors (Fig. 1). The mechanical and functional properties of the lamina vary greatly among cell types depending on the relative ratios of different lamin isoforms, and are related to source tissue stiffness.^35,36^ Cells lacking lamins A and C have fragile nuclei that are more deformable under mechanical strain, exhibit altered mechanotransduction signaling, abnormal condensation of chromatin, an abnormal distribution of nuclear pore complex's (NPCs) and reduced viability.^37-39^ Lamins interact with chromatin either directly or through histones and other lamin-associated proteins including emerin, lamin B receptor (LBR), heterochromatin protein 1 (HP1), barrier-to-autointegration factor (BAF), LEM domain-containing protein 3 (LEMD3), and several lamin associated polypeptide-2 (LAP2) isoforms.^40^ These interactions can occur at the periphery and interior of the nucleus. Tethering of peripheral chromatin to the nuclear lamina occurs in specific genomic regions termed lamina-associated domains (LADs), typified by repressive heterochromatin which reduces transcription factor accessibility resulting in low gene expression levels (Fig. 1).^41,42^ Lamins also impact gene expression through their interaction with transcription factors affecting proliferation, differentiation and apoptosis.^40,43^ Furthermore, mutations in the gene encoding A-type lamins (LMNA) have been associated with at least 8 different diseases collectively termed laminopathies, including Hutchinson Gilford Progeria syndrome and Emery Dreifuss Muscular Dystrophy.^44^ A-type lamins have been linked to the maintenance and regeneration of several mesenchymal tissues and have been proposed to be regulators of mesenchymal stem cell regeneration.^45^ Lamin A and SUN proteins also mediate an interaction with emerin, an integral transmembrane protein which is found on both the ONM and INM. Emerin, like lamin A, has several functions in the nucleus including the indirect regulation of gene expression, RNA processing and chromatin dynamics.^46^

## Nuclear mechanotransduction

While several mechanotransduction pathways have been identified with their origins in the cytoplasm, recent work has identified the existence of mechanotransduction pathways intrinsic to the nucleus. Specifically, the ability of the nucleoskeleton to dynamically remodel in response to applied mechanical stress. Lamin A/C levels have been shown to scale with tissue stiffness, with modulation enhancing substrate stiffness directed differentiation,^36^ suggesting a central role for this protein in mechanotransduction.^47,48^ Buxboim et al. demonstrated that matrix stiffness couples to myosin-II activity to promote lamin A/C dephosphorylation at Ser22.^49^ Lamin A/C is highly phosphorylated during mitosis as the lamina is disassembled to facilitate cell division.^50,51^ On stiff substrates, cell spread area and myosin-II activity are high, resulting in high cytoskeletal pre-stress and flattening of nuclei. This high nuclear pre-stress state is associated with downregulation of lamin A/C phosphorylation, decreasing lamin A/C solubility and strengthening of the lamina (Fig. 1). Lamin A/C reorganisation has been observed in cells exposed to shear stress.^52^ When shear stress was applied to isolated nuclei, a decrease in phosphorylation at Ser390 was also observed, suggesting that lamin A/C conformation is mechanosensitive, with tension suppressing the affinity of phosphorylation associated enzymes.^36^ Similarly, under conditions of high cell contractility with apical actin stress fibers and an intact LINC complex in fibroblasts on rigid substrates and during MSC osteogenesis, Ihalainen et al. showed that the Ig-domain of lamin A/C is more concealed in the basal than apical nuclear envelope, providing additional evidence of a conformational change in lamin A/C under cytoskeletal compressive force.^53^ This is consistent with other findings demonstrating a vertical polarization of lamin A/C in the presence of high cytoskeletal tension and actin cap stress fibers.^54^ Furthermore, the level of lamin A/C drives the translocation of the lamin-promoting transcription factor, retinoic acid receptor γ (RARG), to the nucleus, so that lamin A/C protein levels feedback into lamin A/C transcription.^36,47^

Another INM protein, emerin, interacts with both lamin A/C and chromatin, and also mediates nuclear stiffening in response to mechanical perturbations. Guilluy et al. demonstrated that pulses of force applied via magnetic tweezers to beads attached to nesprin-1 on isolated nuclei could induce emerin mediated nuclear stiffening.^55^ Tyrosine phosphorylation of emerin in response to force triggers a rearrangement of the LINC complex which reinforces its connection with lamin A/C (Fig. 1). Emerin also has a role in mechanosensing through its modulation of nuclear actin polymerisation, which controls nuclear export and transcriptional activity of MRTF-A.^56,57^

Small forces, in the low piconewton range, which are too low to induce protein unfolding may still trigger nuclear mechanotransduction. Local dynamic force applied to integrins and transmitted via an intact and tense actin cytoskeleton to the nuclear envelope has been shown to result in direct displacements of Cajal body-associated protein complexes.^58^ Similarly, chromatin remodelling in response to mechanical perturbation has been demonstrated on timescales preceding MRTF-A nuclear transport, suggesting a direct impact of nuclear deformation on chromatin structure.^59^ Recently, Tajik et al. demonstrated that direct stretching of chromatin leads to transcription upregulation.^60^ Using magnetic twisting cytometry of a bead attached to the plasma membrane, they observed that subsequent transmission of force through the actin cytoskeleton and LINC complex led to chromatin deformation and force-induced upregulation of a GFP-tagged transgene. This mechanoregulation of chromatin dynamics and histone acetylation is likely to be further moderated by lamin A/C level and organization.^61,62^

In addition to direct mechanical perturbations the nucleus also responds to mechano-chemical stimulation via osmotic loading. We have shown that hypotonic challenge induces chromatin expansion and nuclear swelling while hyperosmotic challenge induces rapid chromatin condensation,^63^ which is associated with increased nuclear stiffness.^64^ Enyedi et al. have recently revealed, in zebrafish, a mechanism whereby the nuclear membrane acts to instigate a mechanotransduction pathway directing an inflammatory response to tissue damage.^65^ Tissue damage causes osmotic cell and nucleus swelling at the wound margin. The swelling-induced nuclear membrane stretch activates an inflammatory signaling cascade through altered enzyme-lipid interactions, in a manner mediated by lamin A/C associated nuclear membrane tension.^65^ These studies demonstrate that the nucleus can respond directly to mechanical perturbation, with alterations in both gene regulation and nuclear mechanical properties independent of cytoplasmic biochemical and cytoskeletal responses.

## Nuclear mechanics associated with cell function

The mechanical properties of the nucleus are a function of the nucleoplasm, chromatin, and the nuclear lamina. The chromatin filled nucleoplasm is softer and more viscous than the lamina, and both structures exhibit power-law rheology, lacking any characteristic timescale, when nuclei are subjected either to micropipette aspiration or indented by atomic force microscopy.^66^ The nucleoplasm behaves as a Maxwell material, in that it possesses both viscous and elastic properties, and through particle nano-tracking of a 100 nm bead undergoing Brownian motion, was found to have a Young's modulus of 36 Pa.^67^ To put this into context, bone marrow stiffness ranges from 200–700 Pa, while muscle tissue stiffness is approximately 10 kPa.^36^ However, another study that applied force to a 500 nm bead positioned in the nucleoplasm using magnetic tweezers obtained a Young's modulus of 250 Pa,^68^ suggesting that mechanical properties may depend on the length scale and cell type in which they are measured. The nucleus as a whole, when tested using micropipette aspiration^69,70^ or unconfined compression^71^ exhibits solid viscoelastic behavior with Young's moduli on the kilopascal range; values 1–2 orders of magnitude higher than that of nucleoplasm. The structural stiffness of the nucleus is predominantly derived from the nuclear lamina, with individual components of the nuclear lamina conferring specific mechanical properties. While B-type lamins contribute to nuclear integrity, lamins A and C are the most important contributors to nuclear mechanical properties.^72^ Lamin A/C levels increase with host tissue stiffness, such that cells residing in stiff tissues exhibit high lamin A/C:lamin B stoichiometry and increased nuclear stiffness on micropipette aspiration. Through investigation across a range of cell types, Swift et al. demonstrate that B type lamins dominate the elastic response while lamins A and C dominate the viscous response.^36^ Cells deficient in lamin A/C demonstrate defective nuclear mechanics and mechanotransduction.^37,73^

In addition to lamin A/C, chromatin organization also regulates nuclear mechanics. Induction of chromatin condensation in embryonic stem cells (ESCs) using Ca^2+^ and Mg^2+^ resulted in significantly stiffened nuclei with large decreases in creep compliance.^74^ Heo et al. also demonstrated that chromatin condensation induced in response to hyperosmotic shock or short-term dynamic tensile strain (10 min) in MSCs led to an increase in nuclear stiffness.^64^ In addition to condensation, nuclear stiffness can also be modulated via chromatin tethering to the nuclear envelope. Schreiner et al. demonstrated in fission yeast—which lack a nuclear lamina—that chromatin tethers to the nuclear membrane contribute significantly to nuclear stiffness by restricting chromatin flow in response to cytoskeletal forces *in vivo*, and in isolated nuclei perturbed with optical tweezers.^75^

### Nuclear mechanics and mechanotransduction in disease

Nuclear mechanics and nucleo-cytoskeletal coupling play a key role in cellular mechanosensing. Aberrant nuclear mechanics—often associated with mutations in lamins and LINC complex components—lead to altered nuclear activity, impaired structural dynamics, aberrant mechanosensing and cell signaling which are associated with a growing range of disease scenarios including muscular dystrophy, dilated cardiomyopathy, premature aging, hearing defects and cancer.^76,77^ Mutations in the LMNA gene, which encodes lamins A and C, cause a variety of human diseases termed laminopathies, including Hutchinson-Gilford progeria syndrome (HGPS), dilated cardiomyopathy, limb-girdle muscular dystrophy, and Emery-Dreifuss muscular dystrophy (EDMD).^78^ Mutations in lamin A/C can alter nuclear stiffness and disrupt LINC complex function, causing prominent defects in cardiac and skeletal muscle.^79-81^ For example, the EDMD lamin mutation L535P leads to an increased resistance to strain specifically in muscle nuclei, although this response can be rescued through inhibition of lamin prenylation via depletion of farnesyl diphosphate synthase gene (*fdps-1*).^81^ HGPS is associated with a lamin mutation which increases the nuclear lamina thickness and stiffens the nucleus to reduce nuclear deformation.^82^ Furthermore, sporadic use of this cryptic splice site in lamin A facilitates a slow build-up of features reminiscent of HGPS in otherwise healthy aged cells.^83^

In addition to nuclear stiffness, appropriate nucleo-cytoskeletal coupling is essential for cell migration in a range of processes including development, wound healing, inflammation, and cancer metastasis.^84,85^ Dynamic positioning of the nucleus during migration on 2D substrates *in vitro* requires cytoskeletal forces.^84^ However, the cell and nucleus face additional obstacles to migration in 3D environments where dense fibrous ECM and tight interstitial spaces often create constrictions smaller than the nucleus, so that deformation of the relatively large and stiff nucleus becomes a rate-limiting step.^86^ Nuclear stiffening of cancer cells, through induction of the HGPS lamin mutation, reduces both cell migration, and nuclear deformation in response to micropipette aspiration.^87^ Interestingly, this suggests that aged cells with accumulation of this HGPS associated lamin mutation may resist metastatic cancer migration. Expression of lamin and LINC complex components may be downregulated in cancer.^88^ Recently, in an *in vitro* model of tumor cell migration through confined spaces, depletion of lamin A was observed to increase the incidence of nuclear envelope rupture.^89^ In agreement with this, others have demonstrated that nuclear envelope rupture occurs in regions with reduced or defective lamin B.^90,91^ Such ruptures require proficient DNA repair mechanisms, and the resulting genomic instability may promote cancer progression.^89,92^ Accordingly, targeting of this process may present an opportunity for the development of anti-metastatic drugs. Another nuclear regulated mechanosensing mechanism has recently been implicated in tumor cell invasiveness. Navarro-Lerida et al. have shown that impeded Rac1 nuclear export—which drives nuclear actin polymerisation controlling nuclear shape and organization—alters the cytoplasmic ratio of Rac1 and Rho, increasing cytoplasmic RhoA signaling and driving tumor invasion.^93^

### Stem cell differentiation

Cellular biophysical properties have been shown to provide a biomarker reflecting the differentiation status of both ESCs and adult MSCs.^94,95^ Using digital holographic microscopy (DHM) and an optical laser trap, subcellular structure and compliance of differentiating myeloid precursor cells has been monitored.^94^ A reduction in phase density observed during monocyte and neutrophil differentiation was associated with an increase in cell compliance, while macrophage differentiation was associated with an increase in both phase density and cell stiffness. Using video particle tracking microrheology, Chen et al. showed that osteogenic induction increased both elastic and viscous moduli of differentiating MSCs, while adipogenic induction decreased both moduli.^95^ Alterations to cell mechanics inevitably impact the nucleus, both structurally and mechanically. Again using particle tracking microrheology in stem cells of varying multipotency, Lozoya et al. demonstrate that nuclear shape is a quantifiable discriminant of the mechanical properties of the perinuclear cytoskeleton, such that the relationship between nuclear shape and perinuclear mechanical properties can be used to discriminate between stem cell types.^96^ These findings suggest that structural connections between the nucleus and cytoskeleton exhibit reciprocal mechanical properties. As the stiffest organelle in the cell, the nucleus plays a central role in defining cell mechanics, particularly in stem cells which have a relatively large nucleus:cytoplasm ratio.^97^ ESCs express very low levels of A-type lamins which increase as the cell differentiates.^98,99^ Chromatin is typically diffuse within ESC nuclei, but condenses into higher order structures as cells differentiate.^100^ Both lamin A/C and chromatin condensation contribute to the increased nuclear mechanical properties exhibited by ESCs as they progress toward differentiation.^74^

Throughout the differentiation process, epigenetic modifications accumulate in the genome so that genes associated with pluripotency and self-renewal are silenced in favor of terminally differentiated genetic programs.^101^ These epigenetic modifications are associated with alterations in nuclear architecture and chromatin organization. Live-cell confocal tracking of the nuclear lamina identifies a highly flexible nuclear architecture in mouse ESCs compared with a more frozen chromatin assembly in terminally differentiated primary mouse embryonic fibroblasts.^102^ This flexible ESC nuclear architecture is characterized by correlated spatio-temporal fluctuations in chromatin compaction and nuclear area.^103^ Gene silencing and activation is also often associated with physical movement of specific genes and chromosomes toward the transcriptionally repressive nuclear periphery or toward the center of the nucleus respectively.^104,105^ Recently, Robson et al. have demonstrated that physical recruitment of genes to the nuclear envelope by muscle-specific nuclear envelope transmembrane proteins (NETs) contributes⅓ to ⅔ of a gene's normal repression during myogenesis.^106^ Another recent study in *Caenorhabditis elegans* embryos has identified a nuclear envelope associated chromodomain protein CEC-4, which anchors heterochromatin through recognition of methylated H3K9 to stabilize induced cell fate.^107^ These phenomena illustrate a role for chromatin organization in governing gene expression and highlight the potential for nuclear deformations and shape changes to impact genome function and cell fate.

While ESCs and their nuclei stiffen with differentiation, recent studies have identified 2 distinct mechanical states in pluripotent ESCs.^108,109^ A state of increased mouse ESC stemness—a naïve pluripotency state—has been identified through the inhibition of mitogen-activated protein kinase (MAPK) and glycogen synthase kinase 3 (GSK3).^110^ This is in contrast to culture in the absence of these inhibitors when the pluripotency state is primed for differentiation.^111^ Chalut et al. paired histone modification analysis with optical stretching to show that naïve ESCs have a significantly stiffer nucleus, coupled with a state of increased chromatin condensation when compared with ESCs in the lower nanog expressing primed state.^108^ More recently, Pagliara et al. have further demonstrated that the ESC nuclei in this primed pluripotency state exhibit auxetic mechanical properties: that is they exhibit a cross-sectional contraction when compressed and a cross-sectional expansion when stretched.^109^ This behavior is at least partially driven by chromatin decondensation as the ESC proceeds from a naïve to primed state. This phenomenon has implications for transcriptional regulation and suggests that auxeticity could be a key mechanosensing mechanism in the initial stages of ESC commitment.

While cell and nucleus mechanics change with differentiation, these changes are further compounded by extrinsic mechanical stimuli. Heo et al. have recently shown that tensile strain applied to MSCs, at levels associated with the induction of fibrochondrogenic differentiation, induces rapid chromatin condensation within 10 min., which stiffened MSC nuclei so they were less deformable when cells were stretched.^64^ This mechanical induction of chromatin condensation requires cellular contractility and is mediated by an initial ATP release in response to strain.^112^ While this condensation was transient, dissipating over the course of 3 hours, repeated tensile strain acted to stabilize the condensed chromatin state to establish a mechanical memory in these cells.^64^ This structural encoding of chromatin in the nucleus may sensitize these differentiating MSCs to future mechanical loading events, defining the trajectory and persistence of their lineage specification.

## Interplay between nuclear and cytoplasmic mechanotransduction

Cellular mechanosensing is dependent on the mechanical properties of the cell and its components. As discussed above, the patency of cytoplasmic f-actin and nucleoplasmic lamin A/C networks is critical to normal mechanosensing. Aberrant mechanosensing occurs in disease scenarios where one or both of these cellular compartments exhibits altered mechanics, often stemming from a mutation in a key structural protein. By way of example, Guilluy et al. found that fibroblasts expressing a phosphoresistant emerin mutant exhibited less bundled actin filaments.^55^ This suggests that nuclear adaption to force is critical to actin cytoskeleton organization, demonstrating that structural elements are physically interdependent. Changes in the relative mechanical properties of the cytoplasm and nucleus also occur in differentiation as a cell adapts to perform a new and specific function. As discussed above, the relationship between perinuclear mechanical properties and nuclear shape provides a prospective basis for discrimination between cell types, or assessment of differentiation progression.^96^

**Figure 2.**
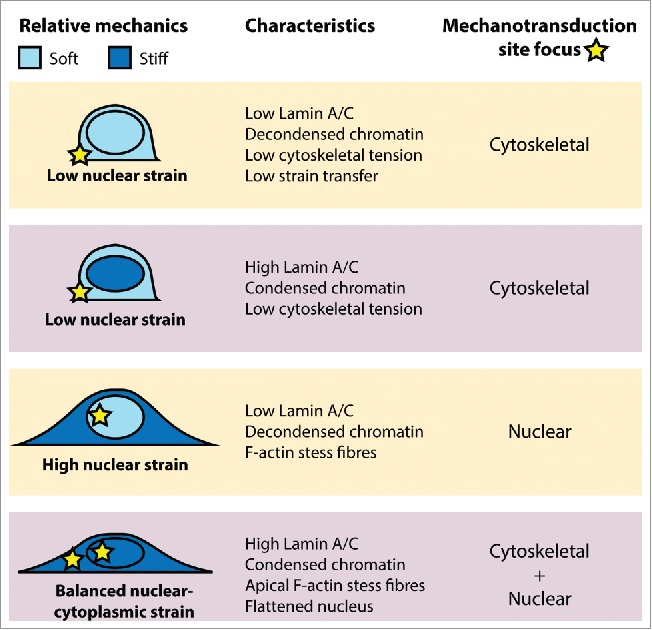
Interplay between nuclear and cytoplasmic mechanotransduction. Schematic illustration depicting relative nuclear and cytoplasmic mechanical properties, and subsequent site specific mechanotransduction in response to an external mechanical stimulus.

We propose that as a cell responds to a mechanical stimulus or dramatically changes its function, as occurs in differentiation and disease, it alters the mechanical properties of both nucleus and cytoskeleton to provide additional sensitivity to specific mechanosensation machinery (Fig. 2). We have demonstrated that multiple episodes of strain application to MSCs sensitize these cells to future mechanical loading events.^64^ Successive episodes of mechanical strain instil a state of chromatin condensation which remains for at least 5 d in the absence of strain. This state of enhanced chromatin condensation likely brings about an increase in nuclear stiffness, which as a result of force balance within the cell, may also increase cytoskeletal pre-stress. This state of enhanced pre-stress may prime specific mechanosensory machinery, both at focal adhesions and in the nuclear interior, to subsequent mechanical perturbations (Fig. 2).

Differential mechanical adaption to direct the source of mechanosensing has been demonstrated elsewhere. Talwar et al. demonstrate that differentiating ESC gene expression is regulated through nuclear mechanical heterogeneity.^113^ Differentiation invoked nuclear stiffening, which was associated with increased lamin A/C expression. When forced to spread on micro-patterned substrates, these lamin A/C mediated changes in nuclear stiffness drive nuclear localization of transcription co-regulator MRTF-A. Driscoll et al. demonstrate that cytoskeletal contractility and connectivity with the nucleus alters nuclear pre-stress and regulates the MSC response to dynamic tensile stretch in terms of another transcription co-regulator: YAP.^114^ The LINC complex was necessary for YAP nuclear translocation in response to tensile stretch.^114^ Another study by Uzer et al. demonstrates that connectivity between the nucleus and cytoskeleton across the LINC complex is critical to the sensation of extremely low magnitude vibratory forces.^115^ Interestingly activation of FAK by high magnitude substrate strain was unaffected by LINC complex decoupling, suggesting that in addition to external mechanical strains, MSCs can also respond internally to vibratory signals enacted through the LINC complex. Indeed, nuclear-cytoskeletal linkages are key to effective crosstalk between the nucleus, cytoskeleton and plasma membrane adhesions.^114,116^

In addition to alteration of transcription regulator nuclear localization, the interplay between cytoskeletal and nuclear mechanics also dictates chromatin dynamics. Makhija et al. demonstrate, using isotropic and elongated cell geometries, a link between cell geometry and chromatin fluctuations where the interplay between active cytoskeletal forces and nuclear rigidity from lamin A/C together regulate nuclear and chromatin dynamics.^62^ Anisotropic nuclear deformation in response to extracellular forces is also regulated by the interplay between cytoskeletal tension, and nuclear architecture including both chromatin and lamin A/C organization.^117^ Together, these studies demonstrate that with alteration of nuclear-cytoskeletal connectivity and mechanics, it is possible for a cell to change the site where a given mechanical stimulus achieves its effect to tailor the mechano-response.

## Conclusions

Recent studies have provided strong evidence that the nucleus acts as a center for mechanotransduction in addition to classical mechanosensors at the plasma membrane. This raises the possibility that the cell, through region specific alterations in mechanical properties, can focus mechanical signals toward a particular mechanosensing apparatus. We propose that with changes in cell function, as occur in disease and differentiation, the nucleus acts as a rheostat to regulate cellular strain distributions by altering its own compliance and resistance to force.

Mechanisms of mechanotransduction in the nucleus are beginning to be unearthed, however, much remains to be discovered in this area. While cell-type specific relationships between nuclear shape and cytoskeletal stiffness have been identified, the mechanisms which differentially regulate nuclear compliance and cytoskeletal tension for a given cell type, or potential mechanosensory focus, remain to be explored. The tools available for studying intracellular biomechanics are improving, and our increased understanding of the connection between physical stresses, nuclear architecture and genome function leads to the recognition of a new realm of cell signaling pathways comprising both biophysical and biochemical events.
